# Supplementary material for: Micro-CT visualization of a promastigote secretory gel (PSG) and parasite plug in the digestive tract of the sand fly Lutzomyia longipalpis infected with Leishmania mexicana
Source: PLoS Negl Trop Dis. 2021 Aug 27;15(8):e0009682. doi: 10.1371/journal.pntd.0009682 (PMC8396784; doi:10.1371/journal.pntd.0009682)
Supplement: Attachment — Submitted filename: Hall et al - Sand fly REVISION - Author Response 05-07-2021.docx [file pntd.0009682.s001.docx]

The Editorial Office

PLOS Neglected Tropical Diseases

5^th^ July 2021

**For attention of:** Guy Caljon (Associate Editor) and Anthony Papenfuss (Deputy Editor)

**"Micro-CT visualization of promastigote secretory gel (PSG) in the digestive tract of the sand fly Lutzomyia longipalpis infected with Leishmania mexicana"**

Dear Editors

We are most grateful for your consideration of our manuscript and we appreciate the thoughtful comments and suggestions made by the two reviewers. Please find below our detailed responses (in itallics and blue font) to each of the suggestions. We believe that the changes have clarified and improved the manuscript and hope that they will enable it to be accepted for publication in your journal.

Thank you for your kind attention.

Yours sincerely,

Martin

Martin Hall

Scientific Associate

Natural History Museum, London, UK

***********************

**Authors Responses**

Reviewer's Responses to Questions

Key Review Criteria Required for Acceptance?
As you describe the new analyses required for acceptance, please consider the following:

Methods
-Are the objectives of the study clearly articulated with a clear testable hypothesis stated?
-Is the study design appropriate to address the stated objectives?
-Is the population clearly described and appropriate for the hypothesis being tested?
-Is the sample size sufficient to ensure adequate power to address the hypothesis being tested?
-Were correct statistical analysis used to support conclusions?
-Are there concerns about ethical or regulatory requirements being met?

Reviewer #1: The authors introduced a new innovative method, the 3D reconstruction using Micro-CT scanning, to visualize infected sand flies.

The Micro-CT scanning was based on a limited sample of 17 sand fly specimens, divided into 5 experimental groups. Therefore, the results are not sufficient for rigorous statistical analyses and the authors correctly used mainly descriptive and exploratory statistics to present and visualize data. Formally, the PCA analysis should be based on higher sample size (at least 30 are recommended). However, the separation of the groups is clear and meaningful (Fig 5). The first two components account for almost 70%, so I agree with the presentation of these data.

Reviewer #2: Methodology is well described and details provided.

--------------------

Results
-Does the analysis presented match the analysis plan?
-Are the results clearly and completely presented?
-Are the figures (Tables, Images) of sufficient quality for clarity?

Reviewer #1: The morphological differences among individual groups (infected flies vs control flies, infected flies with the second bloodmeal vs infected flies without the second bloodmeal) are clearly visible from the figures and well supported with measurements presented in Table 1. The only point at issue may be the nature of the purple stained segments - is it really just PSG? In my opinion, these areas are promastigotes surrounded with PSG and the method does not allow distinguishing how big part corresponds to PSG vs parasites.

*Because we were unable to resolve the morphology of individual parasites we agree with the reviewer that the material, false-coloured in purple was a combination of parasites inside the PSG. To highlight this point, we have redefined what this material represents at the beginning of the results.*

The authors performed the analysis of leishmania numbers in individual groups of infected sand flies (lines 111-114), but the results are not presented.

*We have added this detail about the infections to the opening text of the results.*

Reviewer #2: 4. In line 261: The statement "Based on their morphology, they are predominantly leptomonad promastigotes." Urges for further discussion. Are these parasites the retroleptomonads? What are they doing in the pharynx?

*Sadly, we are unable to differentiate leptomonads from retroleptomonads from the SEM images. According to Serafim et al., this is hard to do through morphology as they appear to be very similar. However, we agree with the reviewer that this possibility requires further comment and have added a few lines to this effect to the discussion.*

5. In the results section, there is no description of the volumes of the abdominal midguts. Anyhow, there is no point of comparing the volumes of abdominal midguts as the blood meals bias the comparisons of uninfected vs infected. In addition, it also skewed the PCA analysis. Therefore, PCA analysis should be done and presented without the abdominal midgut data.

*We agree with the reviewer on this point and have modified the PCA analysis to exclude abdominal midgut data and we include a revised Fig 5.*

8. In figure 1: Please, provide a model without midgut/psg staining

*For comparison with coloured models, a model without false-colour has been added in the new version of the manuscript (Fig 1F) and this is mentioned in the text. However, this none-‘stained’ model was still segmented as no 3D models were generated without segmentation. Images without segmentation are the 2D images of Fig 2.*

9. Figure 2 caption needs to include the differences between time points. T12INF is also in the figure. Should describe what "+" stands for.

*The figure 2 legend has been modified to clarify this.*

10. Figure 5: Needs to include the variance of each component either in the caption or in the graph axis' legends

*The variance of each component has been added to the figure legend.*

--------------------

Conclusions
-Are the conclusions supported by the data presented?
-Are the limitations of analysis clearly described?
-Do the authors discuss how these data can be helpful to advance our understanding of the topic under study?
-Is public health relevance addressed?

Reviewer #1: See the General Comments section

Reviewer #2: Regarding the overall conclusion of the manuscript: What guarantees to the authors that the structures they visualized with micro-CT and named PSG are in fact PSG rather than leishmania parasites? If micro-CT's resolution is not powerful enough to visualize leishmania, how can the authors then postulate that PSG can be visualized in their samples? In Serafim et al, 2018, similar structures encompass massive amounts of parasites in the midgut rather than the light diffraction caused by the presence of massive amounts of fPPG.

*We agree with the reviewer and have modified the reference to the PSG plug by changing it to ‘plug of PSG plus parasites’ and elsewhere throughout the manuscript we refer to the ‘PSG-parasite plug’.*

6. In lines 278-279, the statement "the volumes of the midgut" should be replaced by "the volumes of the thoracic midgut", as that was the region of the midgut significant for the study.

*We have corrected the manuscript here (and elsewhere where any similar clarification was needed) as per the reviewer’s request.*

7. In line 309: The statement "mechanical blockage of the valve by the PSG plug [3]." Should be followed by the sentence "or by the binding of haptomonads to the Stomodeal Valve (Volf et al 2004, Serafim et al, 2018).

*We have modified the sentence to include the requested statement.*

11. In lines 324-330: Are there other technical possibilities of using microCT to visualize Leishmania? Is any kind of leishmania labeling available for this technique? If so, please discuss.

*We have added to the Discussion a consideration of the potential use of synchrotron nanotomography to overcome resolution issues and lengthy scan times at high resolutions. We are not aware of a labelling technique that would function for micro-CT.*

--------------------

<b>Editorial and Data Presentation Modifications?
Use this section for editorial suggestions as well as relatively minor modifications of existing data that would enhance clarity. If the only modifications needed are minor and/or editorial, you may wish to recommend "Minor Revision" or "Accept".

Reviewer #1: Line 41: I suggest adding the taxonomical category (Diptera: Psychodidae) behind Phlebotomine sand flies.

*This has been added – also the Order: Family status of Leishmania.*

Line 53: I suggest omitting the word "clearly" as it is redundant and questionable in case of haptomonads Line 57: I suggest modifying the sentence to: It is the mass of parasites and PSG that obstruct..

*We have removed the word ‘clearly’ and modified the sentence describing the obstruction as suggested.*

Line 185: Here and throughout the text of the manuscript, I suggest to modify the "PSG plug" to just a "plug" (for explanation see General Comments).

*We have modified the text throughout to incorporate this clarification.*

Line 240: stomodeal valve opening diameter

*Text has been corrected to include ‘opening’.*

Reviewer #2: (No Response)

--------------------

Summary and General Comments
Use this section to provide overall comments, discuss strengths/weaknesses of the study, novelty, significance, general execution and scholarship. You may also include additional comments for the author, including concerns about dual publication, research ethics, or publication ethics. If requesting major revision, please articulate the new experiments that are needed.

Reviewer #1:

The manuscript is well written and innovative. It is a pilot study bringing a novel non-invasive method to the research of Leishmania-sand fly interactions. Using Micro-CT, authors visualized the 3D picture of the midgut of sand flies infected with Leishmania parasites in situ for the first time. Although the study is based on limited sample size (understandable for technical reasons), it clearly demonstrated that the gut of infected sand flies was distended in comparison with uninfected control flies and the second non-infected bloodmeal contributed to this distension significantly. Highly valuable are the measurements showing that the opening of the stomodeal valve was significantly higher in infected flies.
I have two objective issues:
. The nature of the plug (purple stained segments): Why authors homologize the plug with PSG only? The substantial part of the plug is formed by Leishmania promastigotes (haptomonads and leptomonads). If authors cannot provide the evidence that the method visualizes the PSG only, then, throughout the manuscript, the plug should be interpret as a formation consisting of both PSG and parasites,
. Authors should provide the numbers of parasites in the studied vector groups.

Reviewer #2:

The manuscript # PNTD-D-21-00697, submitted by Hall and colleagues, describe the establishment of the Micro-CT visualization technique to observe morphological structures of sand flies infected with leishmania. The implementation of technological advances for the study of vector morphology and vector-pathogen interaction needs to be complemented, and by themselves such advances are of interest for the vector biology community. Nonetheless, no evidence is provided that the structures the authors claim to be visualized are in fact PSG. Also, the manuscript needs in multiple instances to include important findings by other authors. Altogether, these issues preclude me to provide a more positive recommendation for the manuscript at this stage. Therefore, my recommendation is for Major Revision.

Major Concerns

1. Line 48: The statement "recent research on an older observation" is merely assumptive - Authors have no evidence that such a research was done based on such an older observation. There was other evidence pointing to the same direction. Statement should be modified.

*We have removed this part of the sentence to reflect that the more recent study of Serafim was not necessarily made in response to the earlier study of Elaiem et al.*

2. Line 57: Regarding the statement "It is the PSG that obstructs the sand fly gut [8,9]" :
a. Statement is misleading. The role of PSG in blocking the sand fly gut is restricted thus far to L. mexicana in Lutzomyia longipalpis. There is no scientific evidence that enough PSG is produced by other species of Leishmania to block their vectors' stomodeal valve.

*We respectfully dispute this – there are a large number of studies which illustrate the presence of a PSG plug in other Leishmania-sand fly combinations to block the stomodeal valve and even in sand fly-midge combinations of the emerging Mundinia subgenera of Leishmania. Also, we have published a number of studies which have looked mechanistically at the role of PSG in both experimental and natural, Old World and New World combinations and have shown that they participate in the behavioural manipulation of the sand fly vector, their transmission and the resulting infections in mice.*

*We accept the earlier point that the micro-CT method cannot differentiate PSG from Leishmania parasites so the obstruction is referred to as the ‘PSG-parasite plug’ throughout the manuscript.*

Authors should either spell out their leishmania and sand fly species in each sentence where they are mentioned or state that such findings about the PSG are yet to be demonstrated in other leishmania-sand fly pairs, and should replace citation (8, 9) by appropriate literature - Schlein 1992, Volf et al 2004

*Rather than repeat the Leishmania and sand fly species throughout the manuscript we have included the following sentence in the discussion, “Studies such as these with other Leishmania-sand fly combinations will prove interesting to elucidate the role of the parasite-PSG plug in vector behaviour and transmission, as the amounts of PSG are likely to vary”.*

b. In Volf et al 2004, it is shown that the attachment of heptomonad-like parasites onto the cuticle liner of the Stomodeal Valve is responsible for the blocking effect, including in Trypanosoma, which has also been noticed in Serafim et al, 2018 and denominated the "Haptomonad Parasite Sphere (HPS)". Paragraph should be modified to incorporate such findings.

*We accept that other authors have shown the presence of haptomonads forms attached to the chitinous surface of the stomodeal valve in a variety of trypanosomatid-vector combinations, including those quoted by the reviewer. The reviewer is asking us to incorporate more recent observations about the nature of the haptomonad plug and the haptomonad parasite sphere, which we are happy to do (please see our modified discussion), but to claim that this is solely responsible for the blocking effect falls prey to the same argument that the reviewer has pointed out to us, namely, that the blocked phenotype is a combination of parasites and PSG.*

c. In Schlein 1991,1992, author(s) showed that the damage of the stomodeal valve by leishmania was responsible for the blockage mechanisms. Authors need to add this information as well.

*We have added Schlein 1991 to the references and in the text.*

3. In line 59: In the statement "so-called "blocked fly hypothesis" [3,10].", the authors mis-cited the studies that postulated the hypothesis, which are Schlein et al. (1991, 1992).

*We thank the reviewer for pointing this mistake out – we have attributed the correct references to this statement.*
--------------------

PLOS authors have the option to publish the peer review history of their article ([https://eur03.safelinks.protection.outlook.com/?url=https%3A%2F%2Fjournals.plos.org%2Fplosntds%2Fs%2Feditorial-and-peer-review-process%23loc-peer-review-history&amp;data=04%7C01%7Cm.hall%40nhm.ac.uk%7C98330192046c40c7945a08d92cdc1b56%7C73a29c014e78437fa0d4c8553e1960c1%7C1%7C0%7C637590146149629241%7CUnknown%7CTWFpbGZsb3d8eyJWIjoiMC4wLjAwMDAiLCJQIjoiV2luMzIiLCJBTiI6Ik1haWwiLCJXVCI6Mn0%3D%7C1000&amp;sdata=bOxpjqOIUgoki7LzLqgqTLTtrKnf8D7apw%2FwdQawntY%3D&amp;reserved=0](https://protect-eu.mimecast.com/s/x7AMCKQDxU8BWlnSvzE6C?domain=eur03.safelinks.protection.outlook.com)). If published, this will include your full peer review and any attached files.

If you choose "no", your identity will remain anonymous but your review may still be made public.

Do you want your identity to be public for this peer review? For information about this choice, including consent withdrawal, please see our Privacy Policy ([https://eur03.safelinks.protection.outlook.com/?url=https%3A%2F%2Fwww.plos.org%2Fprivacy-policy&amp;data=04%7C01%7Cm.hall%40nhm.ac.uk%7C98330192046c40c7945a08d92cdc1b56%7C73a29c014e78437fa0d4c8553e1960c1%7C1%7C0%7C637590146149629241%7CUnknown%7CTWFpbGZsb3d8eyJWIjoiMC4wLjAwMDAiLCJQIjoiV2luMzIiLCJBTiI6Ik1haWwiLCJXVCI6Mn0%3D%7C1000&amp;sdata=x5sj%2F0DcoRIOOgyrERs1KrO0imx3lTCmpKfBHRP4wFY%3D&amp;reserved=0](https://protect-eu.mimecast.com/s/-vAcCLJEyfNm9A1uPKaFy?domain=eur03.safelinks.protection.outlook.com)).

Reviewer #1: No

Reviewer #2: Yes: Iliano V. Coutinho-Abreu
