# Supplementary material for: Micro-CT visualization of a promastigote secretory gel (PSG) and parasite plug in the digestive tract of the sand fly Lutzomyia longipalpis infected with Leishmania mexicana
Source: PLoS Negl Trop Dis. 2021 Aug 27;15(8):e0009682. doi: 10.1371/journal.pntd.0009682 (PMC8396784; doi:10.1371/journal.pntd.0009682)
Supplement: Attachment — Submitted filename: Hall et al - Sand fly REVISION - Author Response 23-07-2021.docx [file pntd.0009682.s002.docx]

The Editorial Office

PLOS Neglected Tropical Diseases

23^rd^ July 2021

**For attention of:** Guy Caljon (Associate Editor) and Anthony Papenfuss (Deputy Editor)

**"Micro-CT visualization of a promastigote secretory gel (PSG) and parasite plug in the digestive tract of the sand fly *Lutzomyia longipalpis* infected with *Leishmania mexicana*"**

Dear Editors

Thank you for your comments on the revision of our manuscript with the revised title as above. We have attended to the new comments and our responses are detailed below.

We hope that these changes meet with your requirements, but please do not hesitate to contact me if you have any further queries about any aspect of our submission.

Thank you for your kind attention.

Yours sincerely,

Martin

Martin Hall

Scientific Associate

Natural History Museum, London, UK

(m.hall@nhm.ac.uk)

***********************

**Authors Responses**

One comment of reviewer 2 remains improperly addressed: "The statement "recent research on an older observation" is merely assumptive - Authors have no evidence that such a research was done based on such an older observation. There was other evidence pointing to the same direction. Statement should be modified." Although authors indicate in the answer letter that this statement was removed, this indicated change was not made in the revised manuscript.

*We apologise to the Editors and Reviewer 2 that this was not in place in the corrected version of the revised manuscript that was submitted. We have now modified the original sentence to reflect that the study of Serafim was not necessarily made in response to the earlier study of Elaiem et al. The new sentence (at lines 51-54 of the re-revised clean text) reads:*

*"Furthermore, it has been shown that second, uninfected blood meals, subsequent to the primary infectious blood meal, promote* Leishmania *replication, amplifying the numbers of parasites by up to 125-fold, thereby increasing sand fly infectivity [4,5]."*

Some minor typographical comments:

Line 99: Le. mexicana -> L. mexicana

*This has been corrected as requested.*

Lines 245, 253: T12 INF -> T12INF

*Thank you for pointing this out – the spaces have been removed in both places as requested.*

Figure Files:

While revising your submission, please upload your figure files to the Preflight Analysis and Conversion Engine (PACE) digital diagnostic tool, <https://eur03.safelinks.protection.outlook.com/?url=https%3A%2F%2Fpacev2.apexcovantage.com%2F&amp;data=04%7C01%7Cm.hall%40nhm.ac.uk%7Cd41d2cbe7936400fda2908d94814eed2%7C73a29c014e78437fa0d4c8553e1960c1%7C1%7C0%7C637620077024924930%7CUnknown%7CTWFpbGZsb3d8eyJWIjoiMC4wLjAwMDAiLCJQIjoiV2luMzIiLCJBTiI6Ik1haWwiLCJXVCI6Mn0%3D%7C1000&amp;sdata=7YJMW35Vawu5V1L8PIsH1hkax52%2F9j6WSGdlUIHZP1s%3D&amp;reserved=0>. PACE helps ensure that figures meet PLOS requirements. To use PACE, you must first register as a user. Then, login and navigate to the UPLOAD tab, where you will find detailed instructions on how to use the tool. If you encounter any issues or have any questions when using PACE, please email us at [figures@plos.org](mailto:figures@plos.org).

*Thank you for the link - we have uploaded all our figures to PACE.*

Data Requirements:

Please note that, as a condition of publication, PLOS' data policy requires that you make available all data used to draw the conclusions outlined in your manuscript. Data must be deposited in an appropriate repository, included within the body of the manuscript, or uploaded as supporting information. This includes all numerical values that were used to generate graphs, histograms etc.. For an example see here: <https://eur03.safelinks.protection.outlook.com/?url=http%3A%2F%2Fwww.plosbiology.org%2Farticle%2Finfo%253Adoi%252F10.1371%252Fjournal.pbio.1001908%23s5&amp;data=04%7C01%7Cm.hall%40nhm.ac.uk%7Cd41d2cbe7936400fda2908d94814eed2%7C73a29c014e78437fa0d4c8553e1960c1%7C1%7C0%7C637620077024934924%7CUnknown%7CTWFpbGZsb3d8eyJWIjoiMC4wLjAwMDAiLCJQIjoiV2luMzIiLCJBTiI6Ik1haWwiLCJXVCI6Mn0%3D%7C1000&amp;sdata=xZeUpCM5xfApvXk0oFQ%2FHAT%2FvpjHGs0jtRH1LhqjOdM%3D&amp;reserved=0>.

*All of our raw numerical data is already included in the Tables within the body of the manuscript.*

Reproducibility:

To enhance the reproducibility of your results, we recommend that you deposit your laboratory protocols in protocols.io, where a protocol can be assigned its own identifier (DOI) such that it can be cited independently in the future. Additionally, PLOS ONE offers an option to publish peer-reviewed clinical study protocols. Read more information on sharing protocols at <https://eur03.safelinks.protection.outlook.com/?url=https%3A%2F%2Fplos.org%2Fprotocols%3Futm_medium%3Deditorial-email%26utm_source%3Dauthorletters%26utm_campaign%3Dprotocols&amp;data=04%7C01%7Cm.hall%40nhm.ac.uk%7Cd41d2cbe7936400fda2908d94814eed2%7C73a29c014e78437fa0d4c8553e1960c1%7C1%7C0%7C637620077024934924%7CUnknown%7CTWFpbGZsb3d8eyJWIjoiMC4wLjAwMDAiLCJQIjoiV2luMzIiLCJBTiI6Ik1haWwiLCJXVCI6Mn0%3D%7C1000&amp;sdata=Iq74peUF2XoKRi0ePE%2BbFH9kgdjCbs1DHYYEbIo4P5M%3D&amp;reserved=0>

*All of our protocols are already published or are reproducible, we believe, from the information contained in the Methods section within the body of the manuscript.*

References

Please review your reference list to ensure that it is complete and correct. If you have cited papers that have been retracted, please include the rationale for doing so in the manuscript text, or remove these references and replace them with relevant current references. Any changes to the reference list should be mentioned in the rebuttal letter that accompanies your revised manuscript. If you need to cite a retracted article, indicate the article's retracted status in the References list and also include a citation and full reference for the retraction notice.

*All of our references have been carefully checked. We have not included any retracted papers nor made any changes to the list in the original revision.*
